# DNA copy number alterations and PPARG amplification in a patient with multifocal bladder urothelial carcinoma

**DOI:** 10.1186/1756-0500-5-607

**Published:** 2012-10-31

**Authors:** Donatella Conconi, Elena Panzeri, Serena Redaelli, Giorgio Bovo, Marco Volante, Paolo Viganò, Guido Strada, Leda Dalprà, Angela Bentivegna

**Affiliations:** 1 Department of Neuroscience and Biomedical Technologies, University of Milan-Bicocca, Monza, Italy; 2 Medical Genetics Laboratory, S. Gerardo Hospital, Monza, Italy; 3 Department of Pathology, S. Gerardo Hospital, Monza, Italy; 4 Department of Clinical and Biological Sciences, University of Turin at San Luigi Hospital, Turin, Italy; 5 Urology Division, Bassini ICP Hospital, Milan, Italy

**Keywords:** Multifocal non-muscle-invasive bladder cancer, Transitional cell carcinoma, Array-CGH, DNA copy number variations, *PPARG*, FISH

## Abstract

**Background:**

Bladder cancer is the seventh most common cancer worldwide and over 90% are transitional cell carcinoma (TCC). At the first time of diagnosis at least 70% of TCC present as superficial bladder cancer. Because the clinical outcome of superficial bladder tumors is relatively unpredictable, there is a pressing need to identify markers that may predict tumor recurrence and progression and new treatment strategies.

**Case presentation:**

We present a unique case of a 67-year old male who underwent total cystectomy after repeated trans-urethral resections of the bladder for multifocal non-muscle invasive bladder cancer. The first and the third tumor were diagnosed as high grade non-infiltrating (HGNI), while the second as carcinoma in situ (CIS). We performed both array comparative genomic hybridization and a targeted chromosomal profile by UroVysion in order to detect copy number variations (CNVs) that may be involved with tumor recurrence and progression. The overall data from this study provide new evidence for the monoclonal origin of urothelial tumor multifocality as several genetic changes were found in different tumors of the same patient. From the analysis of shared CNVs two gained regions emerged at 3p25.2 and 12q23.2, including *PPARG* and *ASCL1* genes, respectively. The copy number level of these genes would seem inversely mutually correlated and highly dependent on histological grade, because the highest level of amplification at 3p25.2 was evidenced in the two HGNI samples, while the highest level of copy number gain at 12q23.2 was reported in the CIS.

**Conclusion:**

We provide new evidence on the role of PPARG in initiation and maintenance of bladder cancer. For the first time we also suggest a possible explanation for the elevated expression of PPARG in this type of tumor through a focal high level amplification at 3p25.2. Furthermore, a new gene, *ASCL1*, emerged as a potential candidate to assist PPARG in bladder carcinogenesis.

## Background

Bladder cancer is the seventh most common cancer worldwide and the fourth most common cancer diagnosed in men in the USA and European countries [1]. Over 90% of bladder cancers are transitional cell carcinoma (TCC), and at the first time of diagnosis, at least 70% of TCC present as superficial tumors confined to the mucosa (pTa disease) or lamina propria (pT1). In the remaining cases, tumors present as muscle invasive disease (≥pT2) with no history of superficial disease. Low-grade pTa tumors have a high risk of recurrence (70%) but rarely progress to muscle-invasive tumors, conversely high-grade pTa and pT1 tumors show a high risk of progression [2].

Because the clinical outcome is relatively unpredictable and the limitations of current therapeutic options, there is a pressing need to identify markers that may predict tumor recurrence and progression and novel treatment strategies.

According to the scoring system established in 2006 by seven European Organisation for Research and Treatment of Cancer trials, the factors predicting the risk of recurrence and progression for non-muscle-invasive bladder cancer (NMIBC) are: *i*) multifocal disease, *ii*) concomitant carcinoma in situ (CIS) and *iii*) tumor size >3 cm [3]. Two theories have been proposed to explain the urothelial tumor multifocality: in the oligoclonal one, the presence of different clones of altered cells give rise to polyclonal tumors (field effect); instead, in the monoclonal theory, the presence of a single altered cell clone that spreads to multiple sites by intraepithelial or intraluminal seeding, gives rise to tumors with shared genetic changes in the same patient [4].

In the present study we analyzed three tumor samples from the same patient who presented multiple recurrences within a short time-span in order to detect copy number variations (CNVs) that may be involved with tumor recurrence/progression and to assess the molecular genetic relationships among the multiple coexisting tumors. The overall data provide new evidence for the monoclonal origin of multifocal disease, as several genetic changes were found in different tumors of the same patient. In particular, one CNV includes the gene for PPARG, a member of the peroxisome proliferator-activated receptor (PPAR) subfamily of nuclear receptors, which resulted highly expressed in bladder cancer and could be an attractive molecular target for the development of novel treatment strategies for this type of tumor [5].

## Case presentation

**Figure 1:**
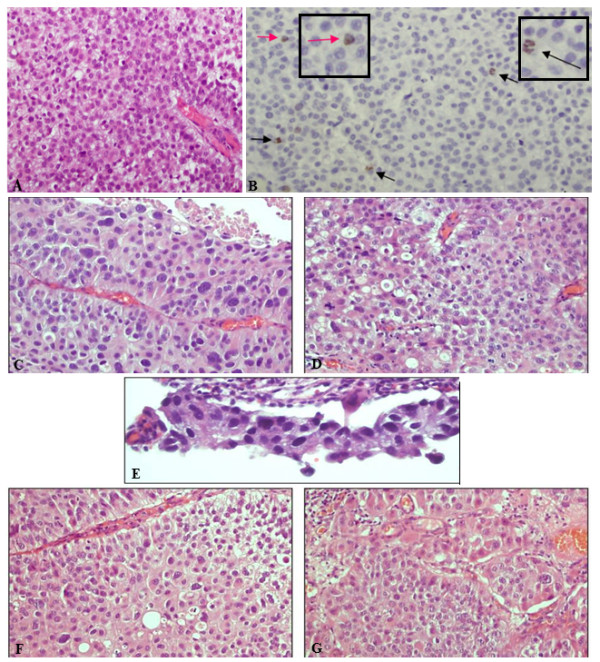
**Representative images of the three TURBs. A**-**D**: First TURB (28CR), high grade transitional cell carcinoma, non-infiltrating. **A**,**C**,**D**: Hematoxylin and eosin staining. **B**: Ki-67 staining; positive nuclei (pink arrows) and metaphases (black arrows); magnification of the images in the boxes. **E**: Hematoxylin and eosin staining of the second TURB (41CR), carcinoma in situ. **F**-**G**: Hematoxylin and eosin staining of the third TURB (3CR), high grade transitional cell carcinoma, non-infiltrating.

We report a unique case of a 67-year old Caucasian male with NMIBC. Three samples were obtained from trans-urethral resections of the bladder (TURB) within nine months: 28CR the first; 41CR, the second, after less than two months; 3CR, the third, after seven months from the second. The first TURB evidenced many lesions projecting into the lumen, with partially solid aspects, occupying the slum, the bladder neck and almost completely the anterior wall and the cuff. The patient underwent antibiotic treatment for urinary infections (TAVANIC 500). After the routinely procession for histological diagnosis, the sections were stained with haematoxylin and eosin and examined by two pathologists expert in urological pathology. The sample was diagnosed as non-infiltrating high grade papillary urothelial carcinoma (HGNI) (according to WHO 2004 classification) (see Figure 1).

The second TURB was performed in the bladder neck, in both lateral wall and in the cuff, the sample was diagnosed as in situ carcinoma (CIS). The patients received intravescical instillation of epirubicin (50mg) and bacillus Calmette-Guérin (BCG), one cycle/week for 6 weeks, according to European Association of Urology (EAU) clinical guidelines [6].

The last TURB was performed with Hexvix-based photodynamic diagnosis and showed hyperemic mucosa, scars of previous endoscopic resections, multicentric recurrence of the right retrotrigonal wall. A complete resection of recurrence, scars and suspected areas was carried out. The sample was diagnosed as HGNI. Finally, the patient underwent total cystectomy.

## Methods

FISH analysis on Formalin-Fixed, Paraffin-Embedded (FFPE) tissue sections was performed using UroVysion bladder cancer kit (Vysis, Wiesbaden, Germany) and Poseidon™ Repeat Free™ *PPARG* (3p25) Break probe (Kreatech Diagnostics, Amsterdam, Netherlands) applying protocols described elsewhere [7]. At least 100 cells for each preparation were scored. The statistical significance of differences between chromosome 3 polisomy and 3p25 amplification was evaluated by Student's *t* test on separate counts of 100 nuclei. Differences were considered as statistically significant with *P*<0.01. All digital images were captured using a Leitz microscope (Leica DM 5000B) equipped with a charge coupled device (CCD) camera and analyzed by means of Chromowin software (Tesi Imaging, Milano, Italy).

For array-CGH analysis, genomic DNA was extracted from fresh biopsies using Wizard genomic DNA extraction kit (Promega, Madison, WI). Sample preparation, hybridization, and analysis were performed using human 8 x 60KCGH microarrays, feature extraction, and CGH analytics software (Agilent, Santa Clara, CA) according to the manufacturer’s instructions. Sex-matched commercial DNA samples (Promega) were used as reference DNA. The arrays were scanned at 2-μm resolution using Agilent microarray scanner and analyzed using feature extraction v10.10 and DNA analytics v6.5 software. Significant chromosomal aberrations have been determined using algorithm ADM-2 (threshold=5; Absolute minimum average log2 ratio=0.25; at least three or more consecutive probe sets). A low-level copy number gain was defined as a log2 ratio >0.25 and a copy number loss was defined as a log2 ratio <−0.25. A high-level gain or amplification was defined as a log2 ratio >1.5 [8]. The presence of mosaicism was calculated as described elsewhere [9].

To analyse which ontology classes were over and under represented among the genes delineated within gain and loss regions detected by array-CGH, the GOstat software (available at http://gostat.wehi.edu.au/) was used based on AmiGO (the Gene Ontology database) version 1.8. [10,11].

Immunohistochemistry was performed on FFPE tissue sections using procedure described elsewhere [12]. Slides were incubated overnight at 4°C, with the mouse monoclonal PPAR-gamma antibody (E-8) sc-7273 (*Santa Cruz Biotechnology*), then stained with an *ABC staining system kit* (*Santa Cruz Biotechnology*). Breast cancer slides were used as positive controls. Human ASCL1 protein was detected by means of a monoclonal antibody (24B72D11.1, diluted 1:150, BD Biosciences, San Jose, CA). Antigen retrieval procedure was performed using microwave heating (three 5 minute passages at 750 Watt) in EDTA buffer (pH 8.0). Immunoreactions were revealed by a dextran-chain (biotin-free) detection system (EnVision, DakoCytomation, Glostrup, Denmark), using 3,3’-diaminobenzidine (DAB, Dako) as a chromogen. A small cell lung carcinoma sample served as positive control. To assess the presence of neuroendocrine phenotype, primary antibodies against chromogranin A (CGA) (Clone LK2H10 + PHE5, diluted 1:200, LabVision Corporation, Fremont CA), synaptophysin (SYN) (Clone SY38, diluted 1:50, DAKO, Denmark), CD56/Neural cell adhesion molecules (NCAM) (Clone 123C3.D5, diluted 1:100, Thermo Fischer Scientific, Fremont CA) were performed. Only for CGA and SYN, antigen retrieval procedure was performed using microwave heating in Target Retrieval Solution Citrate pH 6,0 (DAKO). Immunoreactions were revealed by LSAB+ System-HRP (DakoCytomation, Denmark), using 3,3’-diaminobenzidine (DAB, Dako) as chromogen. A neuroendocrine tumor NET G1 sample served as positive control.

### UroVysion findings

We performed FISH analysis by means of UroVysion test on FFPE specimens from the three TURBs. Since the tumoral area in the second TURB (41CR) was very small in size (Figure 1E), the number of analyzable nuclei was an half in comparison to the other two samples (54 vs 100 nuclei). Significant differences among the three samples were evidenced (*P*<0.05, Student *t* test) for chromosomes 3 and 17, and for 9p21 locus. Conversely, for chromosome 7 a significant difference was observed only between the first and the second biopsy (Table 1).

**Table 1:** Statistical analysis of Urovysion data

**A**		**1^biopsy**	**2^biopsy**	**student*t*test**
**chr3**	mean	5.22	6.22	**p**=**0**,**011**
	d.s.	1.78	3.05	
**chr7**	mean	3.18	3.65	**p**=**0**,**042**
	d.s.	1.29	1.47	
**chr17**	mean	3.67	4.37	**p**=**0**,**008**
	d.s.	1.34	1.88	
**9p21**	mean	1.4	1.89	**p**=**0**,**0017**
	d.s.	0.89	0.92	
**B**		**1^biopsy**	**3^biopsy**	**student*t*test**
**chr3**	mean	5.22	4.1	**p**=**0**
	d.s.	1.78	1.73	
**chr7**	mean	3.18	3.33	p=0,374
	d.s.	1.29	1.08	
**chr17**	mean	3.67	3.07	**p**=**0**,**0007**
	d.s.	1.34	1.11	
**9p21**	mean	1.4	0.99	**p**=**0**,**0012**
	d.s.	0.89	0.86	
**C**		**2^biopsy**	**3^biopsy**	**student*t*test**
**chr3**	mean	6.22	4.1	**p**=**0**
	d.s.	3.05	1.73	
**chr7**	mean	3.65	3.33	p=0,128
	d.s.	1.47	1.08	
**chr17**	mean	4.37	3.07	**p**=**0**
	d.s.	1.88	1.11	
**9p21**	mean	1.89	0.99	**p**=**0**
	d.s.	0.92	0.86	

**A**-**B**-**C**: paired comparison of Urovysion data using student *t* test. Statistically significant differences are in bold (p<0.05).

**Figure 2:**
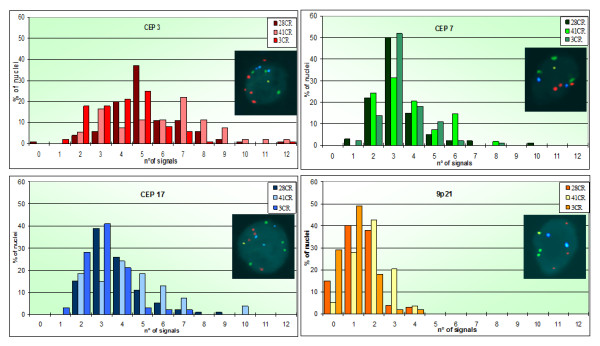
**Urovysion signals distributions.** Urovysion FISH with red (chromosome 3), green (chromosome 7), aqua (chromosome 17) and gold (locus 9p21) probes. Comparison of signals distributions in the three TURBs, with an example of the most representative cell.

Analyzing the signal distribution, i.e. the percentage of nuclei with a specific number of signals for each probe, some similarities can be appreciated between the first and the third sample. For example, we found mostly loss or disomy considering the 9p21 locus, with a very low percentage of nuclei presenting more than 2 signals (7% and 4% respectively, Figure 2); on the contrary, a higher presence of polysomy (24%) was observed in the second biopsy. Furthermore, considering chromosome 3 and 17, the second specimen showed higher percentage of nuclei with polysomy.

### Array-CGH findings

**Figure 3:**
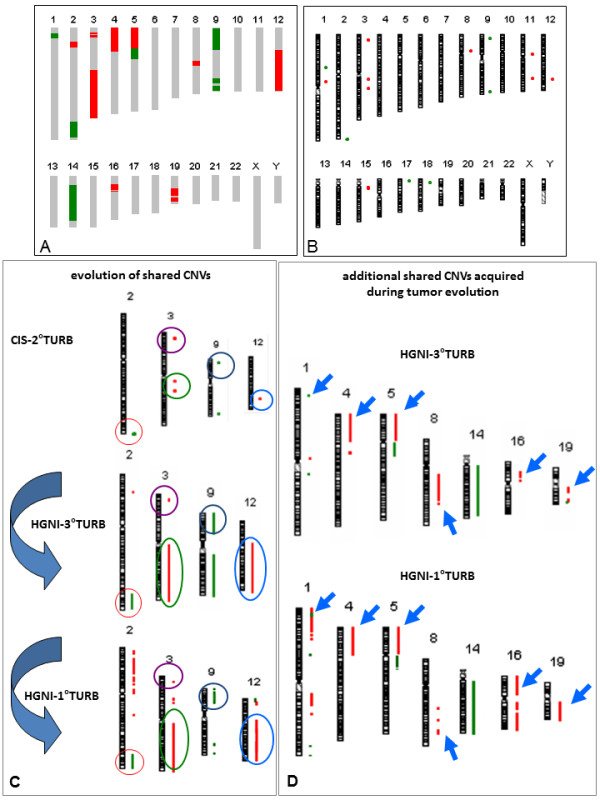
**Array-CGH. A**: Common CNVs in loss (green) and in gain (red) between the first and the third biopsy. **B**: Second biopsy. **C**: Evolution of shared CNVs; each color identifies a specific CNV. **D**: Additional shared CNVs acquired during tumor evolution; the shared CNVs are indicated by arrows.

We also performed array-CGH analysis using genomic DNA extracted from the three tumors. The three biopsies shared 6 CNVs (Figure 3A and B): 4 gains (3p25.2-p25.1, 3q13.11, 3q13.13, 12q23.2), and 2 losses (2q37.3, 9p24.1). The diagram in Figure 3C suggests a possible evolution of the shared CNVs starting from the CIS. To identify possible enrichment of functional groups in the genes included in these regions, a gene ontology annotation analysis was performed using the GOstat software. Interestingly, a statistically significant result emerged only for genes included in the two gained regions 3p25.2-p25.1 and 12q23.2, which include *PPARG* gene and *ASCL1* gene, respectively (Additional file 1: Table S1). In particular, the most representative GO classes were for *fat cell differentiation* regulation. Moreover, between the first and the third TURB, which also shared the same histotype, the number of shared CNVs is significantly increased (Figure 3D and Additional file 2: Figure S1): 12 gains (2p22.3, 3p25.2, 3p25.2-p25.1, 3q11.2-q29, 4p16.3-p11, 5p15.33-p11, 8q22.2-q22.3, 12q13.11-q24.33, 16p12.3-p11.2, 16p11.2-p11.1, 19q12-q13.2, 19q13.33) and 7 losses (1p36.21, 2q35-q37.3, 5q11.1-q13.3, 9p24.3-p13.3, 9q32-q33.2, 9q34.2-q34.3, 14q12-q32.33). In this case, representative GO classes involved mostly *apoptosis* for CNVs in loss, while for CNVs in gain were found *apoptosis*, *cell growth and lipid metabolic process* (Additional file 1: Table S2). We also found a CNV in loss (9q34.3), exclusively shared between the second and the third sample, while another CNV in gain (11p11.2), between the first and the second tumor.

Then, we focused on the two shared CNVs at 3p25.2-p25.1 and 12q23.2. The former spans over 900 Kb and includes six genes: *VGLL4*, *TAMM41*, *SYN2*, *TIMP4*, *PPARG*, *ATG7*. Array-CGH data indicated a focal amplification at the *PPARG* gene locus (log2 ratio >1.5) in at least two tumors. In particular, the first and the third biopsy revealed a log2 ratio of 4.60 and 2.08, respectively, while a low-level copy number gain was identified for the second biopsy (i.e. the CIS), as the log2 ratio was 0.72.

**Figure 4:**
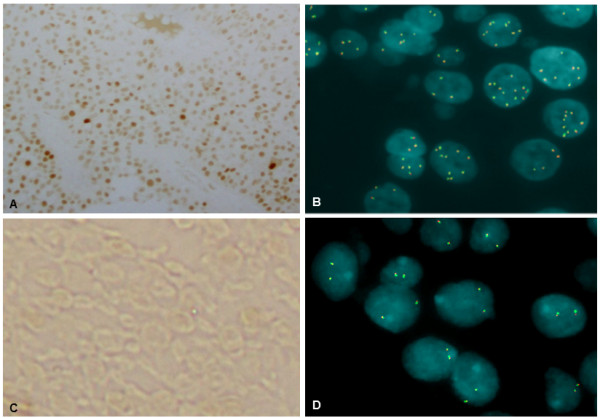
**PPARgamma staining (A-C), and FISH with PPARgamma probe (B-D). A**-**B**: First TURB (28CR). **C**-**D**: normal bladder.

In order to distinguish a polysomy of chromosome 3 from a true amplification, we performed FISH analysis with both Urovysion test assay and the dual-color split probe *PPARG* (Figure 4). We confirmed high level amplification in the first and third sample (*P*<<0.01 Student *t* test), but not in the second (*P*=0.24). We also verified whether the amplification had functional consequences by immunohistochemistry on the first tumor (28CR). PPARG showed an heterogeneous expression: negative tumor cells were mixed with cells at different levels of positivity in the same area (Figure 4A). Furthermore, we assessed that its expression was inversely associated with the expression of Ki67 (Figure 1B), as previously described [13].

The second shared CNV (at 12q23.2) spans 133 Kb and includes *ASCL1* gene, a member of the basic helix-loop-helix (BHLH) family of transcription factors, for which a low-level copy number gain was evidenced in the CIS (log2 ratio of 0.50). Interestingly, the first and the third biopsy shared a more extended region of 86 Mb (from 12q13.11 to 12q24.33), but the averaged log2 ratio was 0.48 and 0.23, respectively. We investigated whether there were consequences at protein level, but no evidence for ASCL1-positive immunoreaction was found. In any case, we cannot exclude that the low-level copy number gain could cause a faint protein expression at least in the CIS (data not shown). Finally, since ASCL1 was indicated as a critical factor involved in neuroendocrine differentiation (NE) [14], we assessed the expression of chromogranin A (CGA), synaptophysin (SYN), CD56/Neural cell adhesion molecules (NCAM), but without positive results.

## Discussion

To date there are two theories to explain the development of multifocal urothelial carcinoma: one suggests a monoclonal origin, while the second a oligoclonal origin [4]. Since the two theories are not mutually exclusive, a detailed characterization and comparison of genetic-genomic alterations of different tumors from the same patient may provide information in this field. We presented a unique case of a 67-year old male with multifocal non-muscle invasive disease: the first and the third tumor were HGNI, while the second tumor was a CIS. Significant differences among all the three tumors were evidenced for 3 of 4 probes of Urovysion (chromosomes 3, 17 and 9p21 locus). The most interesting data emerged from the analysis of shared CNVs among the three tumors: the gain at 3p25.2 and 12q23.2, and the loss at 9p24.1. However, statistically significant ontology classes (*P*<0.05) were evidenced only for the two gained regions, including *PPARG* and *ASCL1* genes, respectively. The former is a member of the peroxisome proliferator-activated receptor (PPAR) subfamily of nuclear receptors that form heterodimers with retinoid X receptors (RXRs) and regulate transcription of various genes associated with immune surveillance, cell proliferation, fatty acid regulation, and angiogenesis [15]. PPARG is a regulator of cell growth, adipocyte differentiation and immune surveillance [16]. Additionally, this nuclear receptor has been implicated in the pathology of numerous diseases including obesity, diabetes, atherosclerosis and in multiple tumor types [17-19]. A possible role for PPARG in bladder cancer has been suggested since it was expressed at higher levels in tumor specimens than in benign urothelium [20]. Furthermore, evidence suggests that this nuclear receptor may either inhibit or facilitate carcinogenesis depending on its level of activation and the specific milieu in which it is acting [5]. Despite this, its potential value for the development of novel targeted treatment strategies was recently emerged [20,21]. For the first time to our knowledge, a molecular justification for the increased expression of PPARG was provided by the presence of a focal high level amplification at 3p25.2, giving also a possible explanation for the monoclonal origin of tumor multifocality, as this CNV was present in 3/3 of the analyzed samples. Because of the limited tumoral area of 41CR sample, we cannot exclude that the lack of high level amplification could be influenced by the surrounding normal tissue, that is revealed by the presence of CNVs in mosaic condition. In addition, since PPARG plays a critical role in the mechanism of action by which bacillus Calmette-Guérin (BCG) inhibits bladder tumor growth [22,23], the decreased level of amplification of the third sample could be explained thanks to the BCG treatment after the second TURB.

The second gene evidenced by GOstat analysis, achaete-scute complex homolog 1 (Drosophila) Human achaete-scute homolog-1 (*ASCL1*), encodes a member of the basic helix-loop-helix (BHLH) family of transcription factors that is expressed in the central and peripheral nervous system during development, and promote early neuronal differentiation. Mis-expression of ASCL1 is correlated with small cell lung carcinomas with neuroendocrine features [24], while its constitutive expression in lung epithelial progenitor cells promotes remodeling and preneoplasia of pulmonary epithelium [25]. ASCL1 was recently involved in neuroendocrine differentiation also in prostate cancer [26]. In this report we associated, for the first time to our knowledge, *ASCL1* gene to bladder cancer. We evidenced a recurrent low-level copy number gain including the locus of *ASCL1* in almost all the tumor samples we examined, especially in the CIS. Even though the three tumors didn't evidence an increased expression of ASCL1, nor neuroendocrine features, we cannot exclude that this genomic imbalance could cause a mis-expression of the protein. It will be interesting to evaluate any possible alterations of ASCL1 mRNA using more sensitive techniques as RT-PCR or RNA-RNA in situ hybridization.

## Conclusions

This is, to our knowledge, the first report that suggests a possible explanation for the elevated expression of PPARG in bladder cancer confirming its critical role in initiation and maintenance of this type of tumor. Furthermore for the first time a new gene, *ASCL1*, emerged as a potential candidate to assist PPARG in bladder carcinogenesis. Finally, this study provides new evidence for the monoclonal origin of the urothelial tumor multifocality, as shared genetic changes were found in the same patient.

## Consent

Written informed consent was obtained from the patient for publication of this Case Report and any accompanying images. A copy of the written consent is available for review by the Series Editor of this journal.

## Abbreviations

ASCL1: Human achaete-scute homolog-1; BCG: Bacillus Calmette-Guérin; BHLH: Basic helix-loop-helix; CCD: Charge coupled device; CGA: Chromogranin A; CIS: Carcinoma in situ; CNVs: Copy number variations; EAU: European Association of Urology; FFPE: Formalin-Fixed Paraffin-Embedded; HGNI: High grade non-infiltrating; NCAM: CD56/Neural cell adhesion molecules; NE: Neuroendocrine differentiation; NMIBC: Non-muscle-invasive bladder cancer; PPAR: Peroxisome proliferator-activated receptor; RT: Room temperature; RXRs: Retinoid X receptors; SYN: Synaptophysin; TCC: Transitional cell carcinoma; TURB: Trans-urethral resections of the bladder.

## Competing interests

The authors declare that they have no competing interests.

## Authors’ contributions

DC and EP carried out all FISH analysis and ontology analysis, immunohistochemistry for PPARG; in addition DC and EP participated in the array-CGH analysis and in the draft of the manuscript. SR carried out all the array-CGH analysis. GB was the responsible for the diagnosis, performed the histopathological staining, took all photomicrographic images and performed the immunohistochemistry for CGA, SYN and NCAM; MV performed the immunohistochemistry for ASCL1. PV and GS were responsible for the surgical and clinical management of the patient. LD participated in the study design and coordination, as well as in revising the manuscript critically. AB coordinated the study and drafted the manuscript. All authors read and approved the final manuscript.

## Supplementary Material

Additional file 1 **Table S1.** Statistically significant ontology classes with p<0.05 from genes included in two CNVs in gain: 3p25.2-p25.1 and 12q23.2. **Table S2.** Statistically significant ontology classes shared between the first and the third biopsy (p<0.05). Click here for file

Additional file 2 **Figure S1.** Array-CGH. **A**: first biopsy. **B**: third biopsy. Click here for file
