# Supplementary material for: DNA copy number alterations and PPARG amplification in a patient with multifocal bladder urothelial carcinoma
Source: BMC Res Notes. 2012 Oct 31;5:607. doi: 10.1186/1756-0500-5-607 (PMC3598781; doi:10.1186/1756-0500-5-607)
Supplement: Additional file 1 — Table S1. Statistically significant ontology classes with p<0.05 from genes included in two CNVs in gain: 3p25.2-p25.1 and 12q23.2. Table S2. Statistically significant ontology classes shared between the first and the third biopsy (p<0.05). [file 1756-0500-5-607-S1.doc]

**Table S1.** Statistically significant ontology classes with p<0.05 from genes included in two CNVs in gain: 3p25.2-p25.1 and 12q23.2.


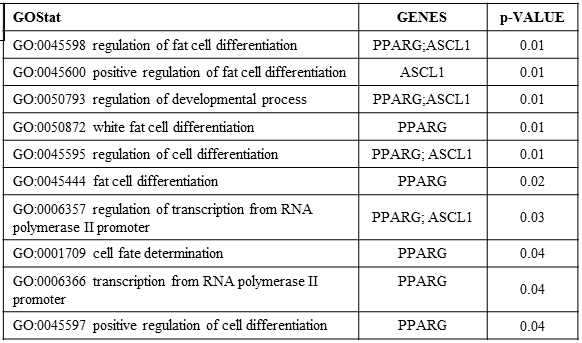


**Table S2.** Statistically significant ontology classes shared between the first and the third biopsy (p<0.05).

| **GAIN** |
| --- |
| GO:0000122 negative regulation of transcription from RNA polymerase II promoter; |
| GO:0001558 regulation of cell growth; |
| GO:0006350 transcription; |
| GO:0006351 transcription, DNA-dependent; |
| GO:0006355 regulation of transcription, DNA-dependent; |
| GO:0006357 regulation of transcription from RNA polymerase II promoter; |
| GO:0006366 transcription from RNA polymerase II promoter; |
| GO:0006629 lipid metabolic process; |
| GO:0006631 fatty acid metabolic process; |
| GO:0006915 apoptosis; |
| GO:0006917 induction of apoptosis; |
| GO:0008219 cell death; |
| GO:0008283 cell proliferation; |
| GO:0008285 negative regulation of cell proliferation; |
| GO:0010467 gene expression; |
| GO:0010468 regulation of gene expression; |
| GO:0012501 programmed cell death; |
| GO:0012502 induction of programmed cell death; |
| GO:0016049 cell growth; |
| GO:0016265 death; |
| GO:0016481 regulation of transcription; |
| GO:0019217 regulation of fatty acid metabolic process; |
| GO:0030154 cell differentiation; |
| GO:0040007 growth; |
| GO:0040008 regulation of growth; |
| GO:0042127 regulation of cell proliferation; |
| GO:0042981 regulation of apoptosis; |
| GO:0043065 positive regulation of apoptosis; |
| GO:0043066 negative regulation of apoptosis; |
| GO:0043067 regulation of programmed cell death; |
| GO:0043068 positive regulation of programmed cell death; |
| GO:0043069 negative regulation of programmed cell death; |
| GO:0045449 regulation of transcription; |
| GO:0045595 regulation of cell differentiation; |
| GO:0045596 negative regulation of cell differentiation; |
| GO:0045834 positive regulation of lipid metabolic process; |
| GO:0045892 negative regulation of transcription, DNA-dependent; |
| GO:0045893 positive regulation of transcription, DNA-dependent; |
| GO:0045923 positive regulation of fatty acid metabolic process; |
| GO:0045941 positive regulation of transcription; |

| **LOSS** |
| --- |
| GO:0000038 very-long-chain fatty acid metabolic process; |
| GO:0001525 angiogenesis; |
| GO:0006357 regulation of transcription from RNA polymerase II promoter; |
| GO:0006366 transcription from RNA polymerase II promoter; |
| GO:0006915 apoptosis; |
| GO:0006917 induction of apoptosis; |
| GO:0008219 cell death; |
| GO:0008283 cell proliferation; |
| GO:0008284 positive regulation of cell proliferation; |
| GO:0008285 negative regulation of cell proliferation; |
| GO:0010468 regulation of gene expression; |
| GO:0012501 programmed cell death; |
| GO:0012502 induction of programmed cell death; |
| GO:0016265 death; |
| GO:0019835 cytolysis; |
| GO:0030154 cell differentiation; |
| GO:0042127 regulation of cell proliferation; |
| GO:0042981 regulation of apoptosis; |
| GO:0043065 positive regulation of apoptosis; |
| GO:0043067 regulation of programmed cell death; |
| GO:0043068 positive regulation of programmed cell death; |
| GO:0045595 regulation of cell differentiation; |
| GO:0045596 negative regulation of cell differentiation; |
