# Supplementary figures and images for: DNA copy number alterations and PPARG amplification in a patient with multifocal bladder urothelial carcinoma
Source: BMC Res Notes. 2012 Oct 31;5:607. doi: 10.1186/1756-0500-5-607 (PMC3598781; doi:10.1186/1756-0500-5-607)

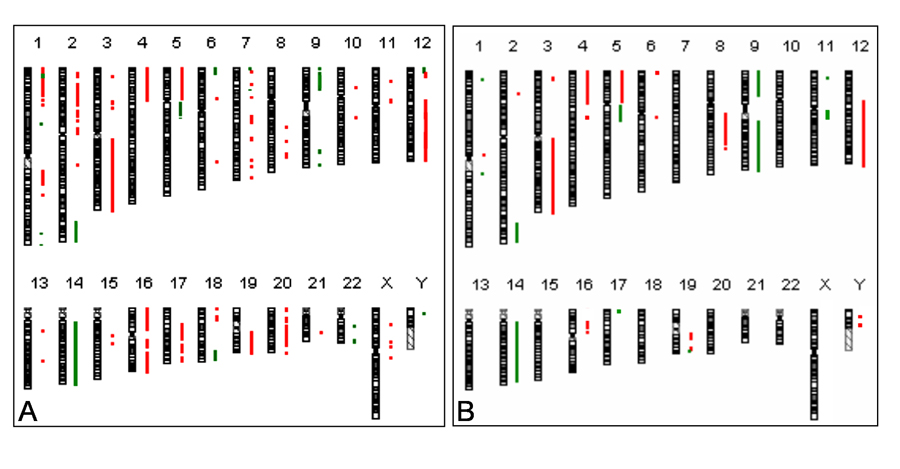

Supplement: Additional file 2 — Figure S1. Array-CGH. A: first biopsy. B: third biopsy. [file 1756-0500-5-607-S2.jpeg]
